# Editorial: Diagnostics and detection of African swine fever virus

**DOI:** 10.3389/fvets.2023.1195138

**Published:** 2023-04-18

**Authors:** Chengjun Zhang, Shuai Li, Mengjia Zhang, Yongtao Li, Luis G. Gimenez-Lirola, Bin Li, Wentao Li

**Affiliations:** ^1^State Key Laboratory of Agricultural Microbiology, College of Veterinary Medicine, Huazhong Agricultural University, Wuhan, China; ^2^Key Laboratory of Prevention & Control for African Swine Fever and Other Major Pig Diseases, Ministry of Agriculture and Rural Affairs, Wuhan, China; ^3^Hubei Hongshan Laboratory, Wuhan, China; ^4^College of Veterinary Medicine, Henan Agricultural University, Zhengzhou, China; ^5^Department of Veterinary Diagnostic and Production Animal Medicine, College of Veterinary Medicine, Iowa State University, Ames, IA, United States; ^6^Institute of Veterinary Medicine, Jiangsu Academy of Agricultural Sciences, Key Laboratory of Veterinary Biological Engineering and Technology Ministry of Agriculture, Jiangsu Key Laboratory for Food Quality and Safety-State Key Laboratory Cultivation Base of Ministry of Science and Technology, Nanjing, China

**Keywords:** African swine fever virus, diagnosis, biomarkers, epidemiology, surveillance

African swine fever virus (ASFV) is a large, enveloped, double-stranded DNA virus that causes a contagious and lethal hemorrhagic disease. Since its first detected in the sub-Saharan Africa, were remains endemic, ASFV rapidly spread to numerous countries including Europe, Asia, and the Caribbean, causing substantial economic losses to the swine industry. Currently, in the absence of commercially available efficacious vaccine, the control of African swine fever (ASF) primarily relies on implementing strict biosecurity measures. One of the key measures is the early and accurate diagnosis of ASF. Therefore, the development of sensitive, rapid, and user-friendly detection methods is crucial. Despite the widespread use of commercialized quantitative real-time PCR (qPCR) and ELISA kits for ASFV detection, many clinical demands remain unmet.

This Research Topic, focused on novel diagnostic technologies for detecting ASFV, with the aim of encouraging new ideas that can improve the prevention and control strategies for ASF. This Research Topic included 13 articles, three of which presented novel ELISA detection methods, three were focused on qPCR methods, two utilized the CRISPR-Cas12a detection system, one evaluated both ELISA and qPCR methods, one article was related to animal experiments, while two discussed sample collection, and one introduced a new sequencing method.

Although there are many ELISA kits available on the market for detecting ASFV antibodies, they mainly target the p72, p30, and p54 proteins (1). However, the discovery of low-virulence, gene-deleted viruses has raised the demand for higher sensitivity and a more comprehensive set of detection targets for ASFV detection.

Jiang et al. developed an indirect ELISA method for identifying wild-type and CD2v-deleted ASFV strains using purified CD2v extracellular domain protein as detection antigen. This method showed excellent specificity to detect CD2v-deleted ASFV, with no cross-reaction with serum infected with other tested swine viruses, high sensitivity allowing identification of ASFV-infected clinical serum samples diluted up to 1:2,560.

Yang Y. et al. utilized the prokaryotic recombinant pB602L protein, a late non-structural protein that displayed strong antigenicity in ASFV, to develop an indirect ELISA method. The results showed that this method exhibited high specificity and sensitivity, with no cross-reactions with other antibodies from other tested swine viruses. This method was able to detect anti-ASFV antibody in serum samples diluted up to 1:6,400 times.

Using prokaryotic expression of proteins p22 and p30, Li J. et al. developed an indirect ELISA kit. Through their investigation, they discovered that optimal signal-to-noise ratios were achieved at various coating volume ratios. The ELISA method they established displayed high sensitivity and could detect positive serum samples at dilutions as high as 1:12,800 times, which was more effective than a commercial kit.

QPCR is the commonly used method for detecting ASFV, which typically employs primer pair and fluorescent probe targeting p72 gene as described in the WOAH Terrestrial Manual (2). Although this method is highly adaptable, it is quite time-consuming. In addition, the emergence of mutated low-virulent strains which induce intermittent detoxification in pigs, as well as the need for gene-deleted vaccine strain development and evaluation, has prompted the investigation of alternative detection techniques.

Hwang et al. developed a fast qPCR method for ASFV detection, which has the advantage of completing the process in just 50 min, a significant improvement compared to conventional PCR assays. The limit of detection (LOD) for genotype II ASFV is 6.91 genomic copies per reaction, while it ranges between 10 and 20 for other ASFV genotypes.

Qi et al. established a qPCR assay based on the ASFV MGF505-7R gene, which has recently been linked to ASFV virulence and could be a valuable target for vaccine development (3). This technique can detect ASFV-infected samples as early as 4 h post-infection, with the sensitivity of up to 10 copies/μL. Furthermore, this method has potential for use in “DIVA” (Differentiating Infected from Vaccinated Animals).

Yang H. et al. established a triplex qPCR method targeting the CD2v and MGF_360-14L and B646L gene of ASFV. The LOD of the method for B646L, MGF_360-14L, and CD2v were 78.9, 47.0, and 82.1 copies/μl, respectively. This technique has proven effective in detecting both genotype I and genotype II ASFV strains and holds potential for the identification of ASFV gene-deleted and wild-type strains.

Due to variations in sensitivity and specificity among different detection methods, it is difficult to confirm whether a sample with low viral load is positive or negative using only one method. Typically, the results are compared to a gold standard reference test. In the study by Schambow et al., a Bayesian latent class analysis (BLCA) model was used to utilized to assess the diagnostic performance of a novel indirect ELISA and a qPCR test for detecting ASFV during a cross-sectional field study in Vietnam. Paired serum and oral fluid (OF) samples were collected from pigs on 30 acutely ASF-affected farms, 37 chronically ASF-affected farms, and 20 unaffected farms, with both assays utilized. The findings indicate that qPCR exhibited superior sensitivity than ELISA for acutely affected pigs, while ELISA sensitivity was higher in the chronically affected pigs. Specificity was nearly 100% for all test/sample types. Additionally, the author compared five parallel testing schemes, which may provide valuable insight for selecting appropriate surveillance strategies.

The Cas12a detection system is a recently developed technology that has been applied to several pathogens. However, due to the limited sensitivity of Cas12a, it is necessary to first employ isothermal amplification methods, such as Recombinase Polymerase Amplification (RPA) or Loop-Mediated Isothermal Amplification (LAMP) amplification to amplify the target gene. The combined use of these methods greatly improves the detection sensitivity and specificity (4). Additionally, the system is cost-effective, does not require expensive amplification equipment, and provides easy-to-read results, which makes it more suitable for clinical applications.

Qin et al. integrated an isothermal LAMP/RPA and CRISPR/Cas12a-mediated cleavage detection system within a single reaction tube, enabling completion of the detection process within 40 min. The results can be visually observed, and the use of a single tube sealed with mineral oil reduces the risk of contamination. Utilizing LAMP as isothermal amplification method and with a reaction time of 60 min, the LOD is 5.8 × 10^2^ copies/μL.

Wang et al. developed a method based on CRISPR-Cas12a combined with G-quadruplex allowing specific detection without the need of fluorescence probes. When paired with RPA amplification, the LOD of ASFV was 10^2^ copies, and the LOD of PCV2 was 10^3^ copies.

To examine blood parameters, viral loads and pathology of the first isolated ASFV in Vietnam, Oh et al. conducted an animal study. The research indicated that the sensitivity to the ASFV varies based on the individual predisposition of the host. The study found that each pig had a different viral load in the blood at the initial time of infection. Group I pigs, which died at 2–5 days post-viremia (dpv), had a higher viral load in the blood at the onset of viremia than Group II pigs, which died at 6–7 dpv. These findings reveal that more genetic and immunological investigations of ASFV-infected pigs are required to elucidate the dynamics of virus susceptibility.

Detecting viruses in water can be challenging due to their typically low concentrations, often falling below the detection limit of qPCR methods. To address this issue, Wu et al. developed a method for detecting ASFV in water by combining of Fe(OH)_3_ modified diatomaceous earth and qPCR. The authors showed that this technique effectively enriched the virus in water samples, increasing LOD in 10 L of ASFV-contaminated water by 1 × 10^4^ times without additional PEG treatment and further improvement with the addition of PEG. This method demonstrated greater sensitivity than the previously reported Al (OH)_3_-modified EGM filter cartridge system and proved capable of detecting low levels of the virus in water samples that tested negative without enrichment.

Collecting samples from suspected ASFV-infected animals through necropsy in pig farms and the wild carries the risk of environmental contamination. Li X. et al. developed a non-invasive method for diagnosing ASFV infection in dead pigs. The authors reported that inguinal lymph node samples, collected using a minimally invasive sampler, contained more ASFV nucleic acids than swabs and constituted an ideal tissue for diagnosing ASFV infection in dead pigs without the need for necropsy.

Genome sequencing is an essential tool for studying viral mutations and tracing their origins. However, traditional methods require the isolation of the virus and ultracentrifugation for enrichment, which can be time-consuming and must be conducted in ABSL-3 laboratory. Zhang et al. established a method for sequencing ASFV positive blood and serum samples without isolating virus. The authors improved C18 spacer MDA (Multiple Displacement Amplification) combined with host DNA exhaustion strategy to remove background DNA and fit Next generation sequencing (NGS) and Third generation sequencing (TGS). Furthermore, they developed a software that enables real-time analysis of TGS depth and coverage by utilizing cloud servers. Using this workflow, they successfully sequenced two uncultured ASFV positive samples in their study.

## Author contributions

CZ, YL, and WL co-wrote the manuscript. All authors contributed to the article and approved the submitted version.
